# *ATG8* is conserved between *Saccharomyces cerevisiae* and psychrophilic, polar-collected fungi

**DOI:** 10.17912/micropub.biology.000446

**Published:** 2021-08-16

**Authors:** Brenna J. Ivory, Hannah M. Smith, Elizabeth Cabrera, Meaghan R. Robinson, Jackson T. Sparks, Amanda Solem, Jun-ichi Ishihara, Hiroki Takahashi, Masaharu Tsuji, Verónica A. Segarra

**Affiliations:** 1 Department of Biology, High Point University, High Point, North Carolina 27268; 2 Department of Biology, Hastings College, Hastings, Nebraska 68901; 3 Medical Mycology Research Center, Chiba University, Chiba 260-8673, Japan; 4 Department of Materials Chemistry, National Institute of Technology (KOSEN), Asahikawa College, Asahikawa, Hokkaido 071-8142, Japan

## Abstract

Autophagy is a conserved catabolic process by which eukaryotic cells respond to stress by targeting damaged or unneeded molecules or organelles for sequestration into specialized vesicles known as autophagosomes. Autophagosomes ultimately facilitate the digestion and recycling of their contents by fusing with the degradative organelle of the cell. Studies of the budding yeast *Saccharomyces cerevisiae* have revealed various types of stress that can regulate autophagy, including starvation and extreme temperatures. While autophagy has not yet been directly shown to confer the ability to survive extreme cold or freeze-thaw stress in yeast, upregulation of autophagy has been directly implicated in the ability of arctic insects to survive cold temperatures. We are interested in investigating the potential role of autophagy in polar habitat survival by cold-loving (psychrophilic) yeast like *Mrakia blollopsis*. To begin to examine the conservation of Atg machinery in polar-collected yeast, we focused on Atg8, a small, ubiquitin-like protein that plays an important role in autophagy. We report that Atg8 is conserved between *S. cerevisiae* and polar-collected yeast, using Atg8 from *Mrakia blollopsis *(strain TGK1-2) as an example. This study represents the first direct examination of autophagy machinery conservation across mesophilic and psychrophilic species of yeast.

**Figure 1. ATG8 is conserved between Saccharomyces cerevisiae and psychrophilic, polar-collected fungi:**
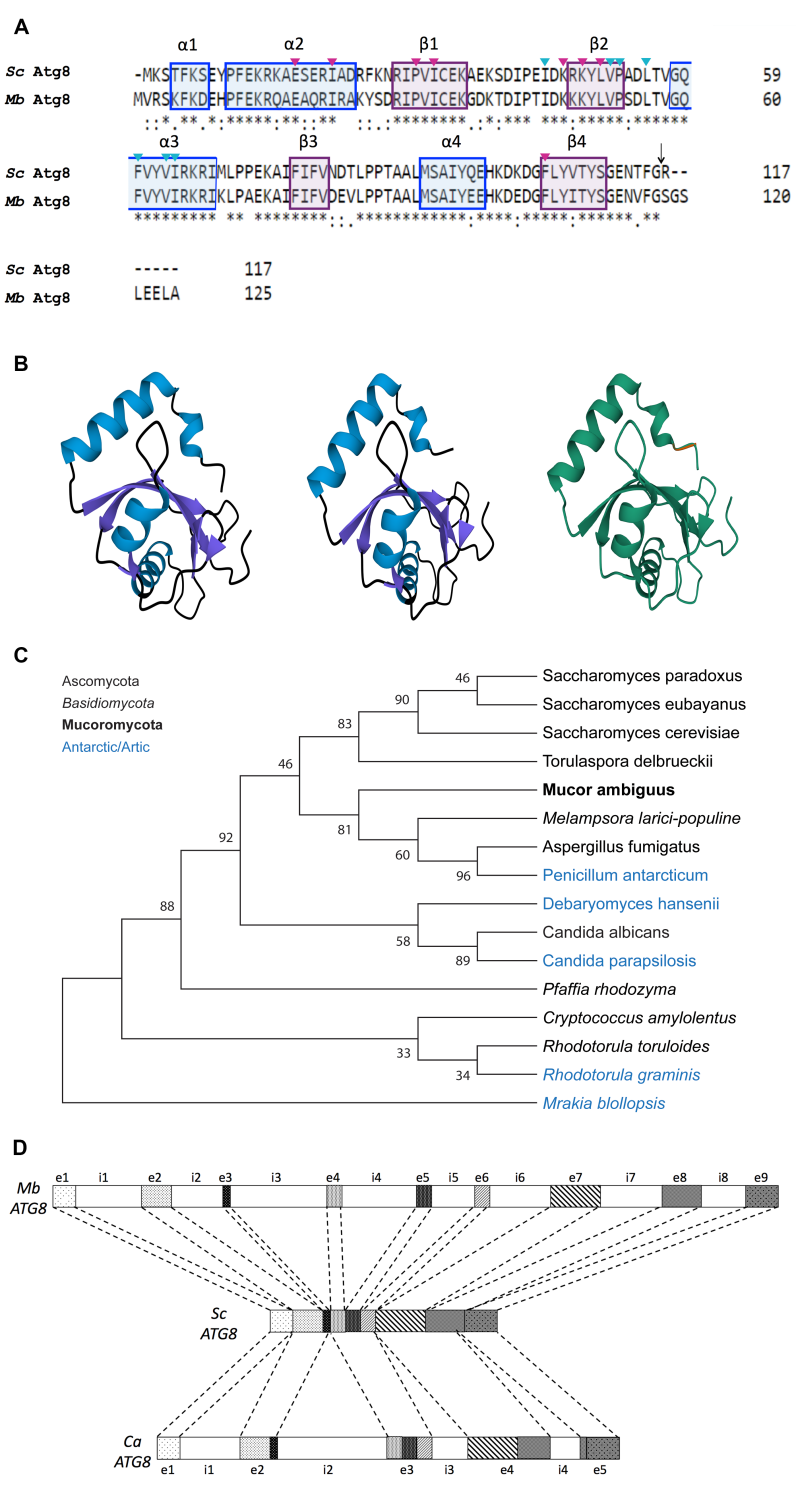
(A) *Atg8 protein sequence is conserved between Saccharomyces cerevisiae (Sc) and the polar collected yeast Mrakia blollopsis (Mb; strain TGK1-2).* Amino acid sequence alignment (Clustal Omega; Sievers *et al.*, 2011) of the full length proteins to highlight overall primary sequence similarity. Known protein structural elements in *Sc* Atg8p are indicated. Within the alignment, amino acids associated with alpha helical or beta-strand secondary structure domains are highlighted in boxes colored blue and purple, respectively. An asterisk (*) indicates a fully conserved residue; a colon (:) denotes conservation of residue side-chain properties; a period (.) indicates residues with side-chains of weakly similar properties; and a dash (-) denotes a gap. The arrow indicates the scissile site within Atg8p, marked by a key glycine residue, at which the C-terminus of the protein can be cleaved and conjugated to the membrane. Residues important for HP1 and HP2 function are marked with magenta and cyan triangles, respectively (Wesch *et al.*, 2020). (B) *Atg8 structure is predicted to be conserved between Sc and Mb.* Phyre2 (Kelley *et al.*, 2015) generated predicted protein structures (100% confidence) for *Sc* Atg8p (left) and *Mb* Atg8 (center). These were aligned using the topology-independent structure comparison algorithm CLICK (Nguyen *et al.*, 2011) to generate a hybrid model (right) that highlights regions of consensus (shown in cyan) non-consensus (shown in orange) (Nguyen *et al.*, 2011). The CLICK hybrid pdb model was visualized using Mol* Viewer (Sehnal *et al.*, 2021). This CLICK structural alignment represented 100% structure overlap and a root-mean-square deviation of atomic positions (RMSD) of 0.0. (C) *Atg8 proteins from polar-collected versus mesophilic yeast do not constitute two distinct clades*. This maximum likelihood tree models the evolutionary relationships between Atg8 amino acid sequences from 16 fungal species (bootstrap 500, MEGA; Kumar *et al.*, 2018). Species known to occur in antarctic and/or arctic regions are shown in blue text. Major divisions within Fungi are noted in the figure key. The evolutionary distance is approximately equal between Atg8 from *Mrakia blollopsis* (bottom) and presumptive orthologues from other species representing the three major divisions of Fungi. Atg8p from *Mrakia blollopsis* does not exhibit a higher degree of conservation with fellow members of Basidiomycota (in italics) nor with other cold-adapted species (in blue font). (D) *Analysis of the Mb (strain TGK1-2) ATG8 genomic sequence compared to cDNA sequence reveals the potential for multiple predicted splicing events within the encoded transcript*. The *Mb (strain TGK1-2) ATG8* genomic sequence contains conserved dinucleotides for splice site definition–GT at the 5’ splice site and AG at the 3’ splice site–suggesting a 9 exon (e) /8 intron (i) gene. The *Mb (strain TGK1-2) ATG8* genomic and protein sequences were compared with their counterparts from *S. cerevisiae (Sc)* and *C. amylolentus (Ca)* to assess potential differences in splicing. Gene sequences were assigned patterns to correspond to exons in the *M. blollopsis* (strain TGK1-2) genomic sequence (exons 1 through 9).

## Description

Autophagy is a stress response mechanism through which eukaryotic cells target and sequester unnecessary or damaged cellular components for degradation and recycling. The hallmark of the autophagy process is the formation of a large (600-900 nm in diameter), double-bilayered autophagosome, a temporary vesicle responsible for sequestering and transporting the cellular components to be recycled to the degradative organelle of the cell. The vacuole functions as the degradative organelle in yeast, where the molecular process of autophagy was first characterized. Autophagy is orchestrated by a group of proteins known as **A**u**T**opha**G**y-related or Atg proteins. Depending on the stress stimuli experienced, eukaryotic cells can deploy a number of different forms of autophagy. These include a general form known as macroautophagy that targets cellular components in bulk for degradation, as well as highly specific forms that target highly specific cellular components such as ribosomes (ribophagy), mitochondria (mitophagy), and peroxisomes (pexophagy). A specific set of core Atg protein machinery is shared among all of these forms of autophagy, and is essential for regulating and bringing about the formation of autophagosomes. Atg8p, coded by the *ATG8* gene in *Saccharomyces cerevisiae*, is one of these core Atg proteins that is essential for autophagy in eukaryotes.

During autophagy, Atg8p undergoes an ubiquitin-like conjugation to the phospholipid phosphatidylethanolamine (PE) on the autophagosome membrane, where it helps recruit cargo and membrane material to facilitate its formation. Mature autophagosomes ultimately fuse with the vacuole, allowing for the recycling of their contents, including Atg8p itself, for degradation (Kirisako *et al.*, 1999; Huang *et al.*, 2000; Nair *et al.*, 2012). In fact, Atg8 is considered as a standard autophagic marker that can be used to track progression of autophagy in cells at the microscopic and molecular levels (Klionsky *et al.*, 2021).

Our understanding of autophagy as a cellular response has heavily relied on studies of the budding yeast *Saccharomyces cerevisiae* as a model system to reveal the molecular underpinnings of the process and the different types of stress that can regulate it, including starvation, exposure to reactive oxygen species, and extreme temperatures. Interestingly, autophagy has not yet been directly linked with the ability to survive extreme cold, despite the fact that freeze-thaw stress has been well characterized in laboratory strains of *S. cerevisiae* and other moderate temperature (mesophilic) yeast. On the other hand, upregulation of autophagy has been directly linked to the ability of an antarctic insect to survive cold temperatures (Teets *et al.*, 2012; Teets and Denlinger, 2013). While autophagy has yet to be directly linked to polar habitat survival by cold-loving (psychrophilic) yeast like *Mrakia blollopsis*, a recent study identified the presence of stress-related genes in other species of psychrophilic yeast (Baeza *et al.*, 2021).

To begin to examine the conservation of Atg machinery in polar-collected yeast, we set out to determine the degree to which the Atg8 protein is conserved between *S. cerevisiae* and species of polar-collected yeast such as *Mrakia blollopsis* (strain TGK1-2). This strain of *Mrakia blollopsis* was isolated from soil samples surrounding the Tokkuri Ike lake in the ice-free Skarvsnes area in East Antarctica (Tsuji *et al.*, 2016; Tsuji *et al.*, 2019). Atg8 was chosen in particular from a larger group of Atg proteins because of its central role in the autophagic cascade of molecular events. Like in *S. cerevisiae*, there appears to be only one *ATG8* gene in *M. blollopsis*, strain TGK1-2.

Protein sequence and structural alignment of Atg8p from *Saccharomyces cerevisiae* (*Sc* Atg8) and Atg8 from the psychrophilic yeast *Mrakia blollopsis* (strain TGK1-2; *Mb* Atg8) shows conservation of secondary structure domains and residues that have been identified as key for Atg8 function (Figure 1A and 1B). Conserved elements in *Mb* Atg8 that are characteristic of Atg8 proteins include the two amino-terminal α helices (α1 and α2) and a C-terminal core that structurally resembles ubiquitin (Noda *et al.*, 2010; Shpilka *et al.*, 2011; Wesch *et al.*, 2020). As in *Sc* Atg8, the C-terminal tail of *Mb* Atg8 contains a conserved glycine residue, which is often referred to as a scissile or catalytic site (Figure 1A, indicated by an arrow). This glycine residue marks the cleavage site at which Atg8 can be conjugated to the membrane or lipidated (Noda *et al.*, 2010; Shpilka *et al.*, 2011; Klionsky and Schulman, 2014; Wesch *et al.*, 2020). Similarly to that of *Sc* Atg8, the ubiquitin-like C-terminus of *Mb* Atg8 consists of a four-stranded β sheet and two α helices (α3 and α4) arranged in a β-grasp fold (Shpilka *et al.*, 2011; Klionsky and Schulman, 2014; Wesch *et al.*, 2020). It is likely that, as in *Sc* Atg8, *Mb* Atg8 serves as a scaffold for protein-protein interactions (Shpilka *et al.*, 2011; Klionsky and Schulman, 2014; Wesch *et al.*, 2020). For example, in characterized Atg8 proteins α1 and α2 align with the ubiquitin-like core, and α3 aligns with β2, to form two deep hydrophobic pockets (HP1 and HP2), respectively. HP1 and HP2 can in turn host protein-protein interactions to additional adaptor and scaffolding proteins (Wesch *et al.*, 2020). Residues known to be important for HP1 and HP2 function in *Sc* Atg8, are also conserved in *Mb* Atg8 (Figure 1A). Given the conservation of key structural and functional elements, we hypothesize *Mb* Atg8 function is similar to that of *Sc* Atg8.

Construction of a maximum likelihood tree to highlight evolutionary relationships between Atg8 amino acid sequences from 16 fungal species (Figure 1C), including species of yeast known to inhabit antarctic and/or arctic environments, shows that the protein is well conserved (Figure 1A-B) despite the evolutionary distance between the Atg8 proteins from *Mrakia blollopsis* (strain TGK1-2) and other forms of cold-loving yeast (Figure 1C).

Furthermore, a search for conserved dinucleotides for splice site definition–GT at the 5’ splice site and AG at the 3’ splice site– revealed that the *Mb* (strain TGK1-2) *ATG8* genomic sequence is likely a 9 exon/8 intron gene (Figure 1D). To assess differences in gene organization and Atg8 proteins across different phyla, *Mb* cold-adapted, mesophilic yeast (*Sc*), as well as fungi with higher intron densities than *Sc* (*C. amylolentus*), the *ATG8* genomic, cDNA, and protein sequences of interest were compared. The *M. blollopsis* polypeptide (strain TGK1-2, 9 total exons) is 69% identical to *S. cerevisiae* and 77% identical to *C. amylolentus*. *S. cerevisiae* (strain ATCC 204508 / S288c, 0 exons) is 69% identical to *M. blollopsis* (strain TGK1-2) and 72% identical to *C. amylolentus*. *C. amylolentus* (CBS 6039, 5 total exons) is 72% identical to *S. cerevisiae* and 77% identical to *M. blollopsis*. The increased complexity in exon/intron gene organization from *Sc* (ascomycota-yeast) to *Mb* (basidiomycota-yeast) is synergistic with previous reports indicating that basidiomycetes such as *Cryptococcus* species display higher rates of alternative splicing (Grutzmann *et al.*, 2014).

All in all, similarities between the Atg8 proteins from *Saccharomyces cerevisiae* and psychrophilic fungi indicate that it is conserved in these organisms. At the same time, some notable differences exist, such as the presence of splicing sites in the *Mrakia blollopsis* (strains TGK1-2) psychrophilic yeast gene. This study represents the first direct examination of autophagy machinery conservation across mesophilic and psychrophilic species of yeast. These findings will serve as a starting point for future investigations to ascertain the extent to which the *function and regulation* of these genes is conserved between mesophilic and psychrophilic yeast.

## Methods

*Sequencing:* The genome of *Mrakia blollopsis* (strains TGK1-2) was sequenced using Pacific Biosciences (PacBio) Sequel*.* Gene prediction was carried out using the Funannotate pipeline.

*Sequence and Structural Alignments:* Clustal Omega (Sievers *et al.*, 2011) was used to perform primary sequence alignments. For structural alignments, the pdb files of the Phyre2-predicted *Sc* Atg8p and *Mb* Atg8 protein structures (Kelley *et al.*, 2015) were aligned using the topology-independent structure comparison algorithm CLICK (Nguyen *et al.*, 2011). A hybrid pdb model was generated that highlights the consensus regions as well as the regions that are predicted to be different (Nguyen, *et al.*, 2011). This hybrid pdb model was visualized using Mol* Viewer (Sehnal, *et al.*, 2021). The CLICK algorithm was also used to obtain analysis parameters such as % structural conservation and root-mean-square deviation of atomic positions (RMSD). Splice sites were identified by aligning the genomic and cDNA sequences (Clustal Omega, EMBOSS Needle) with hand editing based on the presence of conserved splice site dinucleotide sequences (Sievers *et al.*, 2011; Needleman and Wunsch, 1970).

*Phylogenetic Analysis:* The software MEGA was used to construct a maximum likelihood phylogeny with bootstrap support (500 iterations; Kumar *et al.*, 2018). Individual species were chosen for this analysis to include Ascomycota, Basidiomycota and the basal Mucoromycota. Several polar-collected species were chosen to examine potential similarities between cold-adapted fungi from different divisions. Amino acid sequences of presumptive orthologues of the Atg8 protein from *Mrakia blollopsis* were obtained on Genbank (NCBI, 2016). The presumptive orthologue of Atg8 in *Pfaffia rhodozyma* was selected for phylogenetic comparison based on its closest match resulting from BLAST analysis (Altschul *et al.*, 1990).
